# The role of surgery in advanced thymic tumors: A retrospective cohort study

**DOI:** 10.3389/fonc.2022.1073641

**Published:** 2023-01-10

**Authors:** Weifeng Qi, Hui Tian

**Affiliations:** Department of Thoracic Surgery, Qilu Hospital, Cheeloo College of Medicine, Shandong University, Jinan, Shandong, China

**Keywords:** thymoma, thymic carcinoma, surgery, SEER program, prognosis

## Abstract

**Background:**

There is no definitive and detailed treatment guideline for advanced thymic tumors, thus when lymph node and other organ metastasis are present, clinical guidelines recommend chemotherapy-based multidisciplinary treatment. A consensus has been reached that surgery has beneficial effects on partial patients with stage IVA whose metastatic lesions were isolated and resectable, but because of few cases of advanced thymic tumor s and the scarcity of reports, the role of surgery in stage IVB is still unknown. The current study aimed to systematically analyze the role of surgery in advanced thymic tumors based on the Surveillance, Epidemiology, and End Results (SEER) database, with a sufficient number of cases. A secondary aim was to clarify the prognostic value of surgery in advanced thymic tumors.

**Method:**

Data derived from a total of 979 patients with advanced thymoma or advanced thymic carcinoma were collected from the SEER database. Propensity score matching was performed to eliminate confounding factors, and Cox regression analyses were conducted to assess prognoses.

**Results:**

Patients were assigned to four groups based on pathology and whether surgery was performed; thymoma (surgery), thymoma (no surgery), thymic carcinom a (surgery), and thymic carcinoma (no surgery). Disease-specific survival differed significantly in the thymoma (surgery) and thymoma (no surgery) groups, both before and after propensity score matching (both *p* < 0.001). Similarly, disease-specific survival differed significantly in the thymic carcinoma (surgery) and the thymic carcinoma (no surgery) groups (*p* < 0.001 before and *p* = 0.003 after). No total resection, distant metastasis, and thymic carcinoma were all unfavorable prognostic factors.

**Conclusions:**

In the present study surgery had positive effects on advanced thymoma and advanced thymic carcinoma patients who could undergo surgical resection, significantly improving survival times. Total resection of the primary site was the most advantageous form of surgery. The study provides a reference for the clinical treatment of advanced thymic tumors.

## 1 Introduction

Thymic epithelial tumors (TETs) are relatively rare and occur most frequently in the anterior mediastinum. The age of onset of TETs is usually between 50 and 60 years (1, 2). They include two main pathological types; thymoma and thymic carcinoma. Thymomas are divided into five subtypes, A, AB, and B (1–3), and the latter is also known as subtype C (3). The incidence of thymic carcinoma is lower than that of thymoma, but its prognosis is worse, and the 5-year survival rate is approximately 50% (4–6). Surgery is the first-line treatment and main choice for early thymic tumors (Masaoka–Koga I and II). For TETs with Masaoka-Koga III and partial resectable IVA, comprehensive treatment based on surgery is the main treatment (7). There is no definitive and detailed treatment guideline for advanced TETs however, thus when lymph node and other organ metastasis are present the clinical guidelines recommend chemotherapy-based multidisciplinary treatment (7, 8). Previous studies indicate that surgery is beneficial for advanced TETs (9–11), but due to few cases of advanced TETs and the scarcity of reports, the role of surgery is still unknown (12, 13). The present study aimed to systematically analyze the role of surgery in advanced thymic tumors based on the Surveillance, Epidemiology, and End Results (SEER) database with a sufficient number of cases. A secondary aim was to clarify the prognostic value of surgery in advanced thymic tumors.

## 2 Materials and methods

### 2.1 Collection and screening of data

All data were obtained from the SEER database and relevant clinical information was derived from patients with thymic epithelial tumors from 2000 to 2019 (14). The inclusion criteria were (1) A definite diagnosis of thymoma or thymic carcinoma based on pathological or cytological examination (2); thymic tumors of advanced stage (IV)—lymph node or other organ metastasis (3); receiving chemotherapy; and (4) age ≥ 18 years. Patients were divided into four groups based on pathology and whether or not surgery was conducted; thymoma (surgery), thymoma (no surgery), thymic carcinoma (surgery), and thymic carcinoma (no surgery). There was no way to distinguish stages IVA and IVB because the SEER database lacked that information (15).

### 2.2 Statistical analysis

Measurement data are presented as means and standard deviations (SDs) or medians and ranges, and enumeration data are presented as numbers and constituent ratios. Student’s *t*-test was used to analyze measurement data that conformed to a normal distribution and homoscedasticity, otherwise, non-parametric tests were used. The chi-square test and Fisher’s test were used to analyze enumeration data. Propensity score matching (PSM) was performed according to 1:1 nearest neighbor matching with a caliper of 0.03 before each group was compared, to control for confounding factors (16, 17). Survival was analyzed *via* the Kaplan–Meier method. Disease-specific survival (DSS) was the primary endpoint. All data were processed and analyzed using R.

## 3 Results

### 3.1 Baseline characteristics

Based on the above-described criteria a total of 979 patients were identified. The number of patients with thymoma (A, AB, B1, B2, B3) was 12, 21, 36, 57, and 76 respectively and patients with thymic carcinoma were 612. In addition, the more specific pathological types of 165 patients with thymoma were unknown. The count of thymoma(surgery), thymoma (no surgery), thymic carcinoma (surgery), and thymic carcinoma(no surgery) were 173,194,218 and 394 respectively.The proportion of thymic carcinoma is much higher than that of thymoma. There were three different surgical procedures: debulking surgery(thymoma vs thymic carcinoma: 21 cases vs 22 cases), local surgery-removal of only the thymus tumor(thymoma vs thymic carcinoma: 45 cases vs 79 cases), and total/radical surgery-removal of the entire thymus and/or adjacent organs(thymoma vs thymic carcinoma: 107 cases vs 117 cases). Radiotherapy was administered to 52.2% of patient s. The mean age was 56, the median age was 58, and white males were the large st subgroup. Detailed clinical information including tumor history, tumor size, regional metastasis (positive mediastinal lymph node), and distant metastasis (other parts of the lymph node and organ metastasis) is presented in **Table 1**.

**Table 1:** The basic information about thymic tumors.

Variable	No Surgery (N=588)	Surgery (N=391)	Total (N=979)
Age (years)			
Mean (SD)	58.0 (13.9)	53.4 (14.3)	56.2 (14.2)
Median [Min, Max]	59.0 [20.0, 96.0]	55.0 [18.0, 84.0]	58.0 [18.0, 96.0]
Sex			
Female	234 (39.8%)	144 (36.8%)	378 (38.6%)
Male	354 (60.2%)	247 (63.2%)	601 (61.4%)
Race			
White	380 (64.6%)	273 (69.8%)	653 (66.7%)
Other	208 (35.4%)	118 (30.2%)	326 (33.3%)
Pathological Type			
Thymoma(A)	7(1.2%)	5(1.3%)	12 (1.2%)
Thymoma(AB)	7(1.2%)	14(3.6%)	21(2.1%)
Thymoma(B1)	16(2.7%)	20(5.1%)	36(3.7%)
Thymoma(B2)	25(4.2%)	32(8.2%)	57(5.8%)
Thymoma(B3)	21(3.6%)	55(14.1%)	76(7.8%)
Thymoma(NOS)	118(20.1%)	47(12.0%)	165(16.9%)
Thymic carcinoma	394 (67.0%)	218 (55.7%)	612 (62.5%)
Tumor history			
Yes	114 (19.4%)	76 (19.4%)	190 (19.4%)
No	474 (80.6%)	315 (80.6%)	789 (80.6%)
Metastasis level*			
Regional	114 (19.4%)	159 (40.7%)	273 (27.9%)
Distant	474 (80.6%)	232 (59.3%)	706 (72.1%)
Tumor size(mm)			
Mean (SD)	85.4 (71.1)	87.6 (62.2)	86.5 (66.9)
Median [Min, Max]	78.5 [0, 980]	78.0 [11.0, 960]	78.0 [0, 980]
Unknown	226 (38.4%)	49 (12.5%)	275 (28.1%)
Surgery methods			
No	588 (100%)	0 (0%)	588 (60.1%)
Debulking	0 (0%)	43 (11.0%)	43 (4.4%)
Local/Partial	0 (0%)	124 (31.7%)	124 (12.7%)
Radical/Total	0 (0%)	224 (57.3%)	224 (22.9%)
Radiotherapy			
Yes	252 (42.9%)	259 (66.2%)	511 (52.2%)
No	336 (57.1%)	132 (33.8%)	468 (47.8%)
Follow-up time (months)			
Mean (SD)	34.3 (38.5)	56.3 (50.4)	43.1 (44.9)
Median [Min, Max]	20.0 [0, 232]	41.0 [0, 232]	27.0 [0, 232]

*: Regional means mediastinal lymph node metastasis; Distant is other parts of the lymph node and organ metastasis.

### 3.2 Survival analysis

**Figure 1:**
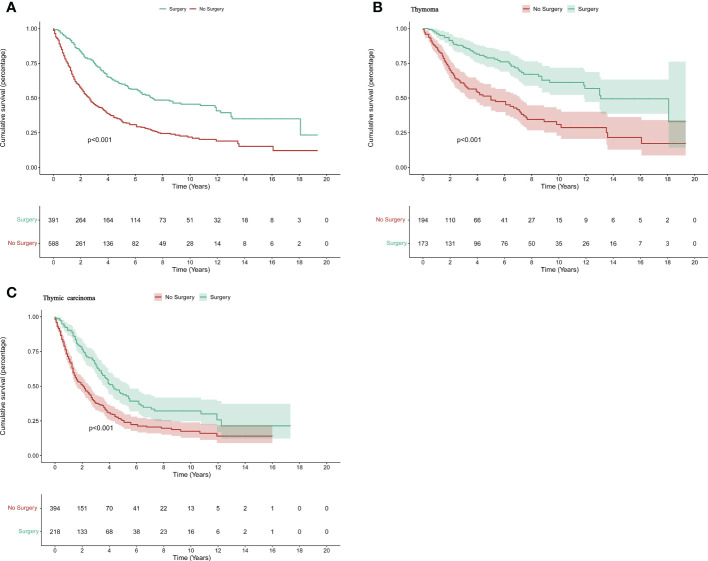
The survival difference in patients with thymic tumors. The K–M curve for all patients **(A)**, patients with advanced thymoma **(B)**, and advanced thymic carcinoma **(C)**.

In 588 patients no surgery was performed and in 391 patients surgery was performed. Disease-specific survival (DSS) time and median survival time(MST) was used to compare the prognosis. Kaplan–Meier and log-rank analyses of the two groups indicated that patients who underwent surgery had a better prognosis (MST: 78 months vs 32 months, *p*<0.001) (**Figure 1A**). Then, the two groups were further divided by pathology, and the same statistical methods were used. The resulting *p* values were all < 0.05, indicating that surgery has a positive effect on prognosis in both thymoma and thymic carcinoma patients(Thymoma_MST: 157 months vs 60 months, *p*<0.001*;* Thymic carcinoma_MST: 51months vs 25 months, *p <*0.001) (**Figures 1B, C**).

### 3.3 PSM

PSM was used in the thymoma and thymic carcinoma groups to reduce the influence of confounding factors and matched variables contained age, sex, race, tumor history, metastasis, tumor size, and radiotherapy. Before PSM, the tumor size, radiotherapy, and metastasis to distant sites had a significant effect on survival. Age and race were confounding factors in the thymoma group and gender was a confounding factor in the thymic carcinoma group (**Tables 2**, **3**). After PSM with 1:1 matching, 91 pairs of patients with thymoma and 146 pairs of patients with thymic carcinoma were successfully matched.

**Table 2:** The information on before and after PSM in patients with thymoma.

Variable	Thymoma (Before PSM)				Thymoma (After PSM)	
	No Surgery N=194	Surgery N=173	*P value*	No Surgery N=91	Surgery N=91	*P value*
Age(years)						
Mean (SD)	58.2 (14.6)	50.0 (13.3)	**<0.001**	53.4 (14.1)	53.7 (13.1)	**0.685**
Median [Min, Max]	59.0 [23.0, 96.0]	51.0 [18.0, 80.0]		55.0 [23.0, 87.0]	55.0 [20.0, 80.0]	
Sex						
Female	85 (43.8%)	78 (45.1%)	**0.807**	39 (42.9%)	42 (46.2%)	**0.767**
Male	109 (56.2%)	95 (54.9%)		52 (57.1%)	49 (53.8%)	
Race						
White	106 (54.6%)	116 (67.1%)	**0.015**	54 (59.3%)	49 (53.8%)	**0.455**
Other	88 (45.4%)	57 (32.9%)		37 (40.7%)	42 (46.2%)	
Tumor History						
Yes	43 (22.2%)	30 (17.3%)	**0.248**	18 (19.8%)	18 (19.8%)	**1.000**
No	151 (77.8%)	143 (82.7%)		73 (80.2%)	73 (80.2%)	
Metastasis*						
Regional	37 (19.1%)	50 (28.9%)	**0.027**	24 (26.4%)	23 (25.3%)	**0.866**
Distant	157 (80.9%)	123 (71.1%)		67 (73.6%)	68 (74.7%)	
Tumor Size						
<=7.5cm	57 (29.4%)	59 (34.1%)	**<0.001**	28 (30.8%)	28 (30.8%)	**0.931**
>7.5cm	65 (33.5%)	92 (53.2%)		41 (45.1%)	43 (47.3%)	
Unknown	72 (37.1%)	22 (12.7%)		22 (24.2%)	20 (22.0%)	
Radiotherapy						
Yes	78 (40.2%)	108 (62.4%)	**<0.001**	47 (51.6%)	50 (54.9%)	**0.656**
No	116 (59.8%)	65 (37.6%)		44 (48.4%)	41 (45.1%)	

*: Regional means mediastinal lymph node metastasis; Distant is other parts of the lymph node and organ metastasis. The meaning of bold values was P value.

**Table 3:** The information on before and after PSM in patients with thymic carcinoma.

Variable	Thymic Carcinoma (Before PSM)			Thymic Carcinoma (After PSM)		
	No Surgery N=394	Surgery N=218	*P value*	No Surgery N=146	Surgery N=146	*P value*
Age(years)						
Mean (SD)	57.9 (13.5)	56.2 (14.4)	**0.168**	57.5 (13.6)	56.4 (14.5)	**0.598**
Median [Min, Max]	59.0 [20.0, 88.0]	58.0 [19.0, 84.0]		58.0 [20.0, 85.0]	58.0 [19.0, 84.0]	
Sex						
Female	149 (37.8%)	66 (30.3%)	**0.061**	44 (30.1%)	48 (32.9%)	**0.614**
Male	245 (62.2%)	152 (69.7%)		102 (69.9%)	98 (67.1%)	
Race						
White	274 (69.5%)	157 (72.0%)	**0.521**	102 (69.9%)	107(73.3%)	**0.517**
Other	120 (30.5%)	61 (28.0%)		44 (30.1%)	39 (26.7%)	
Tumor History						
Yes	71 (18.0%)	46 (21.1%)	**0.353**	28 (19.2%)	30 (20.5%)	**0.769**
No	323 (82.0%)	172 (78.9%)		118 (80.8%)	116(79.5%)	
Metastasis*						
Regional	77 (19.5%)	109 (50.0%)	**<0.001**	46 (31.5%)	44 (30.1%)	**0.799**
Distant	317 (80.5%)	109 (50.0%)		100 (68.5%)	102(69.9%)	
Tumor Size						
<=7.5cm	114 (28.9%)	106 (48.6%)	**<0.001**	58 (39.7%)	63 (43.2%)	**0.836**
>7.5cm	126 (32.0%)	85 (39.0%)		61 (41.8%)	58 (39.7%)	
Unknown	154 (39.1%)	27 (12.4%)		27 (18.5%)	25 (17.1%)	
Radiotherapy						
Yes	174 (44.2%)	151 (69.3%)	**<0.001**	89 (61.0%)	88 (60.3%)	**0.905**
No	220 (55.8%)	67 (30.7%)		57 (39.0%)	58 (39.7%)	

*: Regional means mediastinal lymph node metastasis; Distant is other parts of the lymph node and organ metastasis. The meaning of bold values was P value.

**Figure 2:**
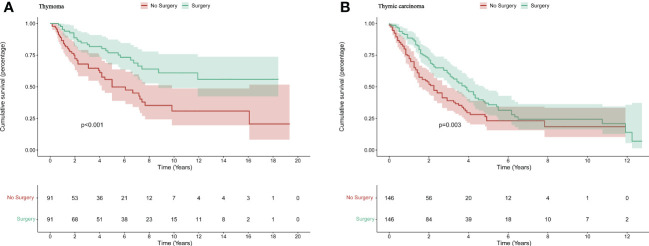
Propensity score matching(PSM): The disease-specific survival for advanced thymoma **(A)** and advanced thymic carcinoma **(B)** after PSM.

Kaplan-Meier and log-rank analyses were then performed to identify further s urvival differences in the thymoma and thymic carcinoma groups. The *p* values were all < 0.05 (Thymoma_MST: NA vs 60 months, *p<*0.001*;* Thymic carcinoma_MST: 46 months vs 27 months, *p <*0.001) (**Figure 2**). Surgery was associated with a favorable prognosis in advanced thymoma patients and advanced thymic carcinoma patients.

### 3.4 Subgroup analysis

**Figure 3:**
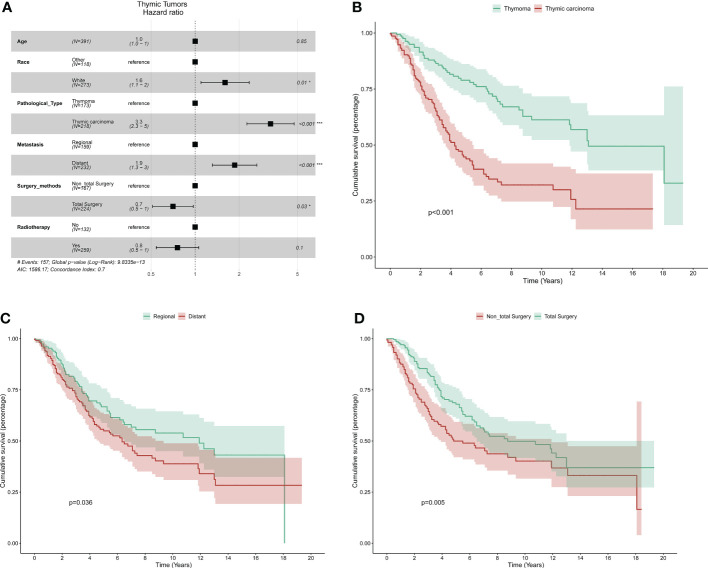
The multivariate cox analysis: **(A)** Forest plot showing prognostic factors for all the patients with surgery. The K–M curve for pathological type **(B)**, metastasis **(C)**, and surgical methods **(D)**.

Cox regression analysis was used to investigate prognostic factors in the 391 patients who underwent surgery. First, univariate Cox analysis was performed separately for age, sex, race, pathological type, tumor history, tumor size, metastasis, surgical methods, lymph node dissection, and radiotherapy. Sex, tumor history, tumor size, and lymph node dissection were not significantly associated with survival, but the rest of the factors listed above were (all *p* < 0.005) (**Table 4**). Multivariate Cox regression analysis was then used to verify the significantly associated factors (**Figure 3A**). Patients with thymic carcinoma had a worse prognosis (hazard ratio [HR] 1.9, 95% confidence interval [CI] 1.3–3.0, *p* < 0.001 for DSS), and metastasis to distant sites was regarded as an adverse factor (HR 0.7, 95% CI 0.5–1.0, *p* = 0.03 for DSS). Total or radical resection of the primary site was associated with better survival (HR 0.7, 95% CI 0.5–1.0, *p* = 0.03 for DSS), and postoperative radiotherapy did not significantly affect survival (HR 0.8, 95% CI 0.5–1.0, *p* = 0.1 for DSS). Survival curves representing pathological type, metastasis, and surgical methods were generated (**Figures 3B** **–D**).

**Table 4:** The results of univariate cox analysis.

Variable	HR	HR.95L	HR.95H	p-value
**Age(years)**	1.0112	0.9996	1.0230	0.0588
**Sex**	0.8767	0.6360	1.2083	0.4214
**Race**	1.6011	1.1026	2.3251	0.0134
**Pathological_Type**	2.8025	1.9834	3.9597	<0.001
**Tumor_history**	0.7009	0.4537	1.0828	0.1092
**Tumor_size**	0.9995	0.9968	1.0022	0.6990
**Metastasis**	1.4132	1.0208	1.9564	0.0372
**Surgery_methods**	0.6405	0.4682	0.8762	0.0053
**LN_Dissection**	0.7984	0.5816	1.0960	0.1637
**Radiotherapy**	0.6881	0.4974	0.9519	0.0240

## 4 Discussion

Thymic epithelial tumors with metastasis to lymph nodes or distant sites have a poor prognosis, and the 5-year survival rate is significantly worse compared to early-stage tumors (4, 18). In previous studies, the respective 5-year survival rates of stage IVA and stage IVB thymic tumors were only 56.3% and 38.2%, and there was an obvious difference in survival between stage IVA and IVB (19). Currently, the clinical treatment for early thymic tumors is clear radical resection. However, the value of surgery in advanced tumors has not been fully verified. Many previous studies explored the role of surgery in thymic tumors with stage IVA and the results showed that surgery could provide long-term survival. So surgery was preferred after deliberative clinic assessments if the tumor could be completely resectable before surgery or after neoadjuvant therapy in thymic tumors with stage IVA (7, 20–24). However, probably due to limited by the number of cases, there are few available reports about IVB thymic tumors. According to a clinical guideline, multidisciplinary discussion was necessary to determine the final treatment plan, and there was insufficient evidence to recommend (7). Meanwhile, a recent treatment guidelines from China stated that standard chemotherapy was the best choice for advanced thymic tumors (8). Hence, it is therefore imperative to define the role of surgery in advanced thymic tumors, which contributes to the formulation of clinical treatment.

In the current study, surgery was associated with significantly better survival time in thymoma patients and thymic carcinoma patients than in the control group. After PSM to reduce confounding factors, these result was further verified. We speculated that surgery played a positive role i n survival by reducing tumor burden and enhancing the effects of chemotherapy. In recent years, the application of surgery in advanced thymic tumors has gradually increased. Hiroyuki et al. reported a case with stage IVB thymic carcinoma that radical resection was performed after concurrent chemoradiotherapy and the patient had 30 months of recurrence-free survival (25); Coincidentally, a clinical team from Japan conducted a cure for a patient with locally recurrent, previously stage IV thymic small-cell carcinoma by surgery combined with perioperative chemotherapy and the tumor did not recur more than 2.5 years (26). Moreover, a retrospective study from Yusuke concluded that surgical intervention was a favorable factor for overall survival in patients with advanced thymic carcinoma (27). Combined with the present study, it is suggested that surgery may improve the survival of patients with advanced thymic tumors.

Furthermore, the resection status of the tumor may affect the patient’s prognosis and a macroscopic complete (R0/R1) was more beneficial to the survival of advanced patients. Markowiak et al. found that the median survival time after R2 resection was 25 months, which was significantly shorter than that after R0 or R1 resection (115 months) (28). Also in the current study, we got a similar result that total resection of the primary site was better than partial resection or debulking surgery concerning survival time. It was well known that R0 resection is almost impossible in patients with lymph nodes or distant metastases. As described in the previous report, a macroscopical R0/R1 resection can be achieved, similar to surgery for malignant pleural mesothelioma, with a significant reduction in tumor burden, which may result in a survival benefit. Interestingly postoperative radiotherapy(PORT) was a positive prognostic factor in univariate analysis, and its statistical significance was eliminated in a multivariate analysis. In fact, there had been many studies on whether postoperative radiotherapy could prolong the survival time of patients with thymic epithelial tumors based on SEER databases. In a study,postoperative radiotherapy had no significant effect on disease-specific survival but had a positive effect on overall survival time in patients with stage IV disease (15). Other studies have yielded different results, and the reason may be that the data processing was different—*i.e.*, putting stages III and IV into one group (29–31). The role of postoperative radiotherapy is controversial in patients with advanced thymic tumors, but some reports have recommended it (32–34). Combined with the current research, considering that advanced thymic tumors cannot be completely resected, postoperative radiotherapy will benefit them. Of course, further studies will be needed to determine the role of postoperative radiotherapy in advanced thymic tumors in the future. Neither tumor size nor lymph node dissection was significantly associated with prognosis. In a multi-institutional analysis, lymph node dissection did not contribute to survival time in patients with thymic malignancies (35). Another article concluded that lymph node dissection was recommended for stage II and higher thymic tumors (36). As mentioned earlier, macroscopic R0/R1 resection was beneficial to the prognosis and coupled with the improved surgical level, it was recommended that positive or suspicious positive nodes should be resected as much as possible although controversial. In the end, thymic carcinoma and metastasis to distant sites were independent adverse factors in this study.

In a recent study based on the SEER database investigating the effects of surgery in Masaoka stage IV thymic carcinoma, it was concluded that surgery positively influenced prognosis (11). Unlike the aforementioned studies and thymic carcinomas, thymomas were also investigated in that study. More cases were analyzed due to an update of the SEER database.

The current study ha dsome limitations. There was no way to distinguish stages IVA and IVB because the SEER database lacked relevant information. We could not acquire detailed information about surgery, such as types of surgical access, resection margins in surgery, and so on. This rendered our findings incomplete and biased. Outcome variables did not include information on tumor recurrence. Lastly, as a retrospective study, while PSM was performed, selection bias strongly influenced the study. Prospective studies are needed for further validation.

In conclusion, in the present study based on the SEER database surgery significantly improved survival time in advanced thymoma and advanced thymic carcinoma patients who could accept surgery. Total resection of the primary site was the most advantageous form of surgery. This study provides a basis for the clinical treatment of advanced thymic tumors.

## Data availability statement

The original contributions presented in the study are included in the article/supplementary material. Further inquiries can be directed to the corresponding author.

## Author contributions

WQ provided the acquisition, analysis, and interpretation of data, statistical analysis, and writing; HT provided the study conception and design. All authors contributed to the article and approved the submitted version.
